# The current and future costs of colorectal cancer attributable to red and processed meat consumption in Brazil

**DOI:** 10.1186/s12913-023-10169-4

**Published:** 2023-10-31

**Authors:** Leandro F. M. Rezende, Thainá Alves Malhão, Rafael da Silva Barbosa, Arthur Orlando Correa Schilithz, Ronaldo Corrêa Ferreira da Silva, Luciana Grucci Maya Moreira, Gerson Ferrari, Paula Aballo Nunes Machado, Maria Eduarda Leão Diogenes

**Affiliations:** 1 Department of Preventive Medicine, Escola Paulista de Medicina, Universidade Federal de São Paulo, São Paulo, Brazil; 2 Instituto Nacional de Câncer, Coordenação de Prevenção e Vigilância, Rio de Janeiro, Brazil; 3 Universidade Federal do Espírito Santo, Programa de Pós-Graduação em Política Social, Vitória, Brazil; 4 Universidad de Santiago de Chile, Escuela de Ciencias de La Actividad Física, El Deport y La Salud, Santiago, Chile; 5 Universidade do Estado do Rio de Janeiro, Instituto de Nutrição, Rio de Janeiro, Brazil

**Keywords:** Cancer, Meat, Red meat, Processed meat, Cost of cancer, Cost-of-illness

## Abstract

**Background:**

Compelling evidence supports the association between red and processed meat consumption and increased risk of colorectal cancer. Herein, we estimated the current (2018) and future (2030) federal direct healthcare costs of colorectal cancer in the Brazilian Unified Health System attributable to red and processed meat consumption. Considering reduced red and processed meat consumption, we also projected attributable costs of colorectal cancer in 2040.

**Methods:**

We retrieved information on red and processed meat consumption from two nationally representative dietary surveys, the Household Budget Survey 2008–2009 and 2017–2018; relative risks for colorectal cancer from a meta-analysis; direct healthcare costs of inpatient and outpatient procedures in adults ≥ 30 years with colorectal cancer (C18-C20) from 2008–2019 by sex.

**Results:**

Attributable costs of colorectal cancer were calculated via comparative risk assessment, assuming a 10-year lag. In 2018, US$ 20.6 million (8.4%) of direct healthcare costs of colorectal cancer were attributable to red and processed meat consumption. In 2030, attributable costs will increase to US$ 86.6 million (19.3%). Counterfactual scenarios of reducing red and processed meat consumption in 2030 suggested that US$ 2.2 to 11.9 million and US$ 13 to 74 million could be saved in 2040, respectively.

**Conclusion:**

Red and processed meat consumption has an escalating economic impact on the Brazilian Unified Health System. Our findings support interventions and policies focused on primary prevention and cancer.

**Supplementary Information:**

The online version contains supplementary material available at 10.1186/s12913-023-10169-4.

## Background

Colorectal cancer is the third most common cancer for both men and women in Brazil, with an estimated 40,990 newly diagnosed cases in 2021, approximately 10% of all cancers (excluding non-melanoma skin cancer) [1]. By 2040, colorectal cancer is expected to increase by over 74% due to demographic changes (population growth and aging) [2]. The projected burden of colorectal cancer suggests an escalating economic impact on the treatment and care costs the Brazilian Health System will have to cope with.

The Brazilian Health System has a coexistence of public, private, and army systems. The Brazilian Unified Health System (Sistema Único de Saúde Brasileiro – SUS) is the most extensive public health system in the world benefiting approximately 200 million inhabitants. Over 70% of Brazilians rely exclusively on SUS [3]. The SUS Outpatient Information System (SIA/SUS) and the Inpatient Information System (SIH/SUS) are nationwide sources of direct healthcare costs and provide publicly available and deidentified inpatient and outpatient care data. In 2018, the federal government spent approximately US$1.7 billion on direct healthcare costs of cancer in Brazil, of which 15% (US$269 million out of US$1.7 billion) were due to colorectal cancer [4].

Primary cancer prevention is pivotal in cancer control, particularly considering the limited access to affordable and effective preventive screening and cancer treatment in low- and middle-income countries. In principle, colorectal cancer is one of the most preventable common cancers. In Brazil, approximately half of colorectal cancer cases and deaths could be prevented or postponed through primary prevention strategies focused on lifestyle risk factors, including diet and nutrition [5].

The International Agency for Research on Cancer (IARC) [6] and the World Cancer Research Fund, and the American Institute for Cancer Research (WCRF/AICR) [7] consider the evidence that processed meat consumption increases the risk of colorectal cancer as sufficient/convincing. This classification is based on consistent evidence showing that consumption of processed meat increases the risk of colorectal (increased risk per 50 g increase in consumption per day) and robust evidence for mechanisms operating in human [7]. Red meat consumption has been classified as probably carcinogenic to humans [6, 7]. WCRF/AICR [7] and the Brazilian National Cancer Institute (INCA) [8] recommend limiting red meat consumption to no more than three portions per week (< 500 g/week). For processed meat, the recommendation is to eat very little, if any. Despite these recommendations, per capita meat consumption has increased [9–11]. The average global consumption of all meats in 2010 was 122 g/day, with high-income and Latin American countries, including Brazil, eating the most [12]. Quantifying the current and future economic burden of colorectal cancer attributable to red and processed meat consumption in Brazil may help inform potential policy and prevention initiatives.

In this study, we estimated the current (2018) and future (2030) federal direct healthcare costs of colorectal cancer in the SUS attributable to red and processed meat consumption. In addition, we estimated potential savings in federal direct healthcare costs of colorectal cancer in 2040 by considering different counterfactual scenarios of reduction in red and processed meat consumption to be achieved in 2030.

## Methods

### Data and study design

This study applied a top-down costing approach and performed a macrosimulation model to estimate the future costs of colorectal cancer attributable to red and processed meat consumption, using the Brazilian population as a case study. We used the following data: 1. Relative risks (RR) from WCRF/AICR meta-analyses [13]; 2. Prevalence data (%) of red and processed meat consumption in adults aged 20 years or older who relied exclusively on the public health system from representative national surveys; 3. Nationwide registries of federal direct healthcare costs of inpatient and outpatient procedures in the SUS in adults aged 30 years or older with cancer. The parameters used in the model are available in Supplementary Material A.

We estimated the potential impact of red and processed meat on federal direct healthcare costs of cancer, assuming a 10-year time lag between exposure and outcome via comparative risk assessment. We used the potential impact fraction (PIF) equation and the Monte Carlo simulation method to estimate the attributable costs and their 95% uncertainty intervals, considering the theoretical-minimum-risk exposure and other counterfactual (alternative) red and processed meat consumption scenarios. We assessed cancer costs attributable to red and processed meat, multiplying PIF by the direct healthcare costs of cancer.

### Relative risk estimates and cancer sites

We considered in our study only colorectal cancer as there is strong evidence of association (convincing or probable) with red and processed meat according to the WCRF/AICR [7]. The WCRF/AICR method of grading evidence has been designed to operationalize the criteria identified by Bradford Hill as contributing to an inference of causation from observational data. Strong evidence of association sustains a judgment of a convincing causal (or protective) relationship that justifies making recommendations designed to reduce cancer risk. This evidence is robust enough to be unlikely to be altered in the foreseeable future as new evidence accumulates [7]. We detailed the list of the 10^th^ Revision of the International Statistical Classification of Diseases and Related Health Problems (ICD-10) codes in Supplementary Material B.

We obtained the ${RR}_{x}$ for colorectal cancer incidence by sex from the WCRF/AICR dose–response meta-analysis [13], considering the increment of $x$ g/day of red and processed meat ($x$=100 and 50, respectively). It is important to enphazise that this meta-analysis [13] represents the last update from WCRF concerning the evidence from observational studies (cohort, nested case–control and case-cohort designs) on the association between foods, nutrients, physical activity, body adiposity and the risk of colorectal cancer in men and women. The WCRF/AICR dose–response meta-analysis is part of the CUP (Continuous Update Project), a global network of researchers that evaluates cancer prevention research.

We converted these measures per increment of 1 g /day of the exposure (${RR}_{1}$) using the following equation [14]:

$${RR}_{1}=exp(\frac{\mathrm{log}({RR}_{x})}{x}).$$

To obtain the RR for each exposition category (RRc) (Supplementary Material C), we used the following equation [15]:

$${RR}_{c}={RR}_{1}^{{M}_{c}-ref},$$

where Mc represents the median value in each category, and ref represents the reference category value (< 70 g/day for red meat and 0 g for processed meat). The reference category reflected the theoretical minimum risk exposure level [7] and the Brazilian INCA recommendations [8].

### Assessment of red and processed meat consumption prevalence

We obtained an estimated prevalence of red and processed meat consumption in the adult population aged ≥ 20 years from the National Household Budget Survey (Pesquisa de Orçamentos Familiares – POF), a cross-sectional nationally representative survey conducted in Brazil in 2008–2009 [16] and 2017–2018 [17]. We considered only adults aged 20 years or older who reported not having health insurance to obtain the prevalence and 95% confidence interval for each red and processed meat consumption by sex.

**Fig. 1:**
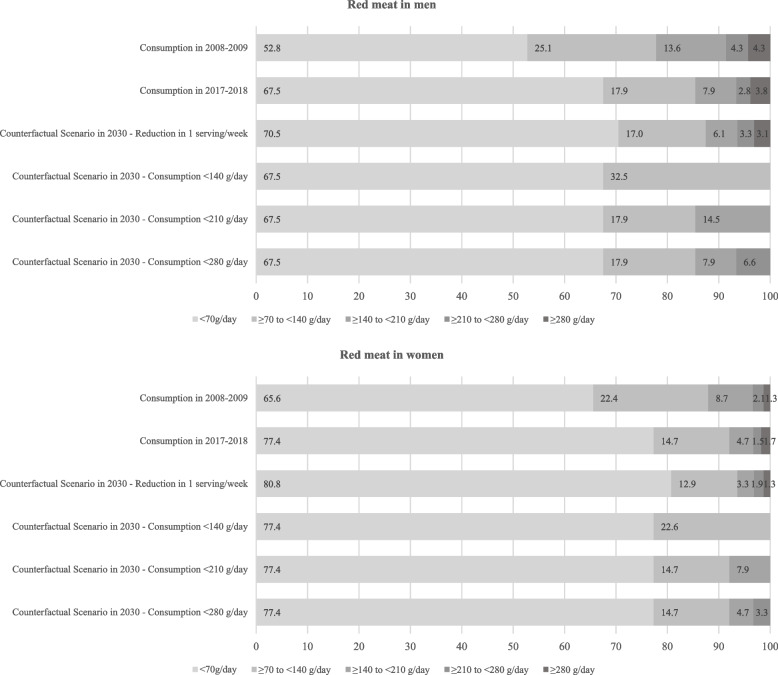
Red meat consumption distribution in 2008–2009 and 2017–2018 and levels fixed in counterfactual (alternative) scenarios to be achieved in 2030

**Fig. 2:**
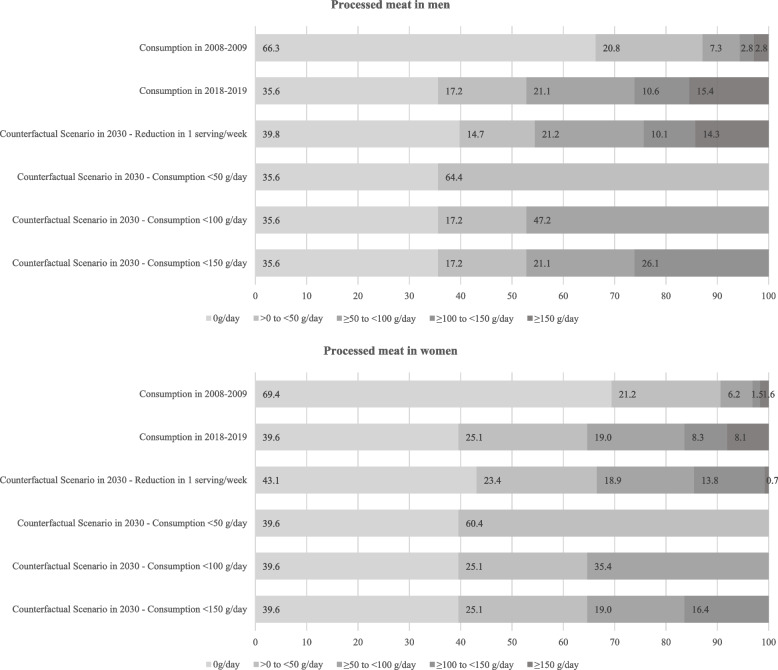
Processed meat consumption distribution in 2008–2009 and 2017–2018 and levels fixed in counterfactual (alternative) scenarios to be achieved in 2030

POF collected two 24-h real-time food records from 34,003 participants in 2008–2009 [16] and 37,690 participants in 2017–2018 [17]. Using a food portion table, we converted reported food amounts into grams [18]. We estimated red meat consumption (g/day) based on the consumption of all types of meat from mammals, such as beef, horse, goat, lamb, mutton, and pork, whereas processed meat (g/day) on the consumption of meat preserved by smoking, curing, salting, the addition of chemical preservatives (e.g., bacon, chorizo, corned beef, ham, pastrami, salami, and sausages). We displayed categories of red and processed meat consumption in grams/day in Figs. 1 and 2, respectively. The reference category (< 70 g/day of red meat and 0 g/day of processed meat) aimed to reflect the theoretical minimum risk exposure level [7] as well as the recommendations of the Brazilian INCA [8].

The POF microdata is available in the public domain via the Brazilian Institute of Geography and Statistics (IBGE) at http://www.ibge.gov.br (Supplementary Material D). We incorporated the complex sample design into all estimates using RStudio version 1.4.1103.

### Counterfactual (alternative) scenarios for red and processed meat consumption

We proposed four counterfactual (alternative) scenarios of population-wide reduction in red and processed meat consumption (observed in 2017–2018) to be achieved in Brazil in 2030 to save direct healthcare costs with cancer in 2040 (Figs. 1 and 2). The counterfactual scenarios considered reduction in one serving per week (Scenario 1: -120 g/week of red meat and -50 g/week of processed meat) and shifting red and processed meat categories, which considered a scenario where everyone consumes: for red meat: Scenario 2) < 140 g/day; Scenario 3) < 210 g/day; Scenario 4) < 280 g/day; for processed meat: Scenario 2) < 50 g/day; Scenario 3) < 100 g/day; Scenario 4) < 150 g/day.

### Federal direct healthcare costs of cancer in the Brazilian SUS in 2030 and 2040

We retrieved registries of federal direct healthcare costs of inpatient and outpatient cancer-related procedures between 2008 and 2019 from the SIH/SUS and SIA/SUS (Supplementary Material D**)**. We used the 10^th^ Revision of the International Statistical Classification of Diseases and Related Health Problems (ICD-10) codes for recovering cancer procedures from information systems (Supplementary Material B). We stratified the direct healthcare costs by sex. Assuming a 10-year time lag between exposure and outcome, we considered the procedures approved for payment in adults with cancer aged 30 years or older in 2030 and 2040.

We performed a simple linear regression to predict the future costs of cancer (dependent variable) as a function of time (independent variable) up to 2030 and 2040 based on the values practiced over time between 2008 and 2019. Controlling for potential confounders while examining the possible determinants of cost is crucial. Once our outcome was the direct healthcare costs over time, it was unnecessary to control for confounders because we observed their effect in the observed costs used to fit the regression model [19]. We transformed the monetary values in Brazilian Real (R$) to United States Dollar (US$), considering the purchasing power parity (PPP) of 2018 (conversion factor 2.226) to current costs and of 2019 (conversion factor 2.281) to future costs [20].

#### Cancer costs attributable to red and processed meat consumption

Based on the abovementioned intermediate inputs of the models, we calculated the (PIF) for colorectal cancer by sex and counterfactual (alternative) scenario using the following equation [21]:

$$PIF= \frac{{\sum }_{i=1}^{n}{P}_{i} {RR}_{i}-{\sum }_{i=1}^{n}{P^{\prime}}_{i} {RR}_{i} }{{\sum }_{i=1}^{n}{P}_{i }{RR}_{i}},$$

where ${P}_{i}$ is the proportion of the population at the level $i$ of red and processed meat consumption in a given year, ${P{\prime}}_{i}$ is the proportion of the population at the level $i$ of red and processed meat consumption in a given counterfactual (alternative) scenario, and ${RR}_{i}$ is the RR of colorectal cancer at the level $i$ of red and processed meat consumption. We displayed the levels $i$ for red and processed meat consumption in Figs. 1 and 2. Of note, the PIF equals the Population Attributable Fraction (PAF) when the counterfactual (alternative) scenario represents the theoretical minimum risk exposure level [21, 22].

To estimate the fraction of colorectal cancer costs attributable to combined red and processed meat consumption, we used the joint PIF/PAF equation [23], which assumes the absence of interaction between risk factors:

$$Joint PIF=1-{\prod }_{i=1}^{n}(1-{PIF}_{i}).$$

To assess the cancer costs attributable to red and processed meat, we multiplied PIF by the total colorectal cancer costs. We considered the prevalence in 2008–2009 and 2017–2018 and the costs of cancer in 2018 and 2030, respectively, assuming at least a 10-year time lag between exposure and outcome (i.e., based on the average follow-up time of prospective cohort studies [13]). Finally, we calculated the potential savings in cancer costs in 2040 if reduced red and processed meat consumption occurred in Brazil to levels fixed in the counterfactual (alternative) scenarios in 2030.

We quantified the uncertainty in all modeled estimates using the Monte Carlo simulation approach [24, 25] with 10,000 iterations. The simulation works thoroughly, producing a draw from the distributions of a) baseline prevalence per red and processed meat consumption category considering a binomial distribution; b) the log of the RR per exposure category for the association of red and processed meat consumption with colorectal cancer risk assuming a normal distribution. We calculated PIF by sex for the 50^th^, 2.5^th^, and 97.5^th^ percentiles as the central estimate and 95% uncertainty intervals across all simulations. Negative values of PIF derived from the Monte Carlo simulation were rounded to 0, assuming that reducing red and processed meat consumption values may not increase the risk of cancer and, consequently, the attributable costs. We used R Studio version 1.3.1093 for analysis.

## Results

### Consumption of red and processed meat in Brazil in 2008–2009 and 2017–2018

Comparing 2008–2009 and 2017–2018, the prevalence of ≥ 70 g/day of red meat consumption (i.e., above the Brazilian INCA recommendations) decreased from 47.2% to 32.5% in men and 34.4% to 22.6% in women. We observed changes in other categories of higher consumption of red meat. For instance, the prevalence of ≥ 280 g/day of red meat consumption changed from 4.3% to 3.8% in men and 1.3% to 1.7% in women (Fig. 1). On the other hand, between 2008–2009 and 2017–2018, processed meat consumption in Brazil increased from 33.7% to 64.4% in men and 30.6% to 60.4% in women. The prevalence of ≥ 150 g/day of processed meat increased from 2.8% to 15.4% in men and 1.6% to 8.1% in women in the same period (Fig. 2).

### Federal direct healthcare costs of colorectal cancer in Brazil in 2018 attributable to the consumption of red and processed meat in 2008–2009

In 2018, federal direct healthcare costs of colorectal cancer were approximately US$ 245 million. Colorectal cancer costs were higher in men (US$ 124 million) than in women (US$ 121 million). We estimated that 3.5% or US$ 8.5 million direct healthcare costs of colorectal cancer were attributable to red meat consumption and 5.1% or US$ 12.6 million to processed meat consumption. The combined fraction of direct healthcare costs of colorectal cancers attributable to combined red and processed meat consumption was 8.4%, corresponding to US$ 20.6 million in 2018 (Table 1).

**Table 1:** Current and future colorectal cancer costs in Brazil attributable to red and processed meat consumption

	**2018**			**2030**		
**Exposure/ sex**	**Cancer costs (US$)**^**a**^	**PIF (%)**^**b**^	**Attributable costs (US$)**	**Cancer costs (US$)**^**c**^	**PIF (%)**^**b**^	**Attributable costs (US$)**
**Red meat**						
men	123,700,380	4.5 (2.9 to 6.0)	5,544,957 (3,575,363 to 7,407,952)	228,906,203 (203,142,066 to 254,670,339)	3.3 (1.9 to 4.7)	7,513,365 (4,286,994 to 10,731,288)
women	121,230,895	2.4 (1.0 to 3.8)	2,940,784 (1,212,365 to 4,608,717)	220,929,379 (195,113,232 to 246,745,527)	1.8 (0.5 to 3.0)	3,886,036 (1,136,571 to 6,529,009)
both	244,931,274	3.5 (2.0 to 4.9)	8,485,740.82 (4,787,727 to 12,016,668)	449,835,582 (398,255,297 to 501,415,867	2.5 (1.2 to 3.8)	11,399,400 (5,405,564 to 17,251,296)
**Processed meat**						
men	123,700,380	5.9 (4.5 to 7.2)	7,254,857 (5,526,420 to 8,946,090)	228,906,203 (203,142,066 to 254,670,339)	19.9 (18.4 to 21.3)	45,517,852 (42,209,324 to 48,788,288)
women	121,230,895	4.4 (3.1 to 5.7)	5,331,079.13 (3,730,607 to 6,938,755)	220,929,379 (195,113,232 to 246,745,527)	14.4 (12.9 to 15.9)	31,798,418 (28,400,492 to 35,106,488)
both	244,931,274	5.1 (3.8 to 6.5)	12,585,935.60 (9,257,027 to 15,884,845)	449,835,582 (398,255,297 to 501,415,867	17.2 (15.7 to 18.7)	77,316,264 (70,609,816 to 83,894,776)
**Red and Processed meat**						
men	123,700,380	10.1 (7.3 to 12.8)	12,536,415 (8,992,399 to 15,794,064)	228,906,203 (203,142,066 to 254,670,339)	22.5 (20.0 to 25.0)	51,503,896 (45,781,241 to 57,226,551)
women	121,230,895	6.7 (4.1 to 9.3)	8,115,681 (4,932,885 to 11,254,349)	220,929,379 (195,113,232 to 246,745,527)	15.9 (13.3 to 18.4)	35,127,771 (29,383,607 to 40,651,006)
Both	244,931,274	8.4 (5.7 to 11.0)	20,652,096 (13,925,284 to 27,048,413	449,835,582 (398,255,297 to 501,415,867)	19.3 (16.7 to 21.8)	86,631,667 (75,164,848 to 97,877,556)

Between parenthesis: 95% uncertainty intervals

^a^Observed direct healthcare costs (inpatient and outpatients) of colorectal cancer in adults ≥ 30 years

^b^Potential impact fraction considering the theoretical minimum risk exposure level

^c^Projected direct healthcare costs (inpatient and outpatients) of colorectal cancer in adults ≥ 30 years

### Projected federal direct healthcare costs of colorectal cancer in Brazil in 2030 attributable to red and processed meat consumption in 2017–2018

In 2030, we projected approximately US$ 450 million in direct healthcare costs for colorectal cancer in Brazil. We estimated that 2.5% or US$ 11.4 million in direct healthcare costs of colorectal cancer would be attributable to red meat consumption and 17.2% or US$ 77.3 million to processed meat consumption. The combined fraction of direct healthcare costs of colorectal cancers attributable to red and processed meat consumption will be 19.3%, corresponding to US$ 86.6 million in 2030 (Table 1).

### The potential impact of the reduction in the consumption of red and processed meat on projected direct healthcare costs of colorectal cancer in Brazil in 2040

We displayed in Figs. 1 and 2 the potential reductions (counterfactual scenarios) in Brazil’s red and processed meat consumption to be achieved in 2030. We estimated that approximately US$ 2.2 to 11.9 million in 2040 could be saved by reducing red meat consumption in 2030. Reducing processed meat consumption to levels fixed in the counterfactual scenarios in 2030 could save US$ 13.7 to 74.4 million in 2040 (Table 2).

**Table 2:** Impact of red and processed meat consumption reduction on colorectal cancer in Brazil in 2040

Exposure/ sex	Projected costs (US$)^a^	Potential saving in colorectal cancer costs (US$) in 2040			
		**Scenario 1 **^**b**^	**Scenario 2 **^**c**^	**Scenario 3 **^**d**^	**Scenario 4 **^**e**^
**Red meat**					
male	317,429,968 (283,179,564 to 351,680,371)	1,285,611 (0 to 5,714,548)	8,148,736 (3,644,857 to 12,421,262)	4,350,948 (0 to 8,865,388)	2,462,824 (0 to 6,860,784)
female	305,064,350 (270,744,803 to 339,383,896)	914,947 (0 to 4,575,602)	3,795,489 (0 to 7,492,750)	1,842,296 (0 to 5,519,205)	894,872 (0 to 4,594,416)
both	622,494,317 (553,924,368 to 691,064,267)	2,200,588 (0 to 10,290,150)	11,944,224 (0 to 19,914,012)	6,193,244 (0 to 14,384,593)	3,357,796 (0 to 11,455,199)
**Processed meat**					
male	317,429,968 (283,179,564 to 351,680,371)	3,827,726 (0 to 9,506,488)	45,175,624 (40,088,556 to 49,928,476)	30,504,042 (25,359,232 to 35,602,440)	16,789,772 (11,243,520 to 22,084,760)
female	305,064,350 (270,744,803 to 339,383,896)	9,840,991 (4,515,539 to 13,923,995)	29,222,740 (24,337,652 to 33,949,132)	16,525,969 (11,294,418 to 21,542,936)	8,037,038 (2,681,575 to 13,122,396)
both	622,494,317 (553,924,368 to 691,064,267)	13,668,717 (2,527,097 to 24,430,482)	74,398,368 (64,426,212 to 83,877,608)	47,030,012 (36,653,652 to 57,145,380)	24,826,812 (13,925,095 to 35,207,156)

Between parenthesis: 95% uncertainty intervals

^a^Projected direct healthcare costs (inpatient and outpatients) of colorectal cancer in adults ≥ 30 years in 2040

^b^Average reduction in one serving per week of red (-120 g/week) and processed (-50 g/week) meat consumption

^c^Everyone consumes < 140 g/day of red meat and < 50 g/day of processed meat

^d^Everyone consumes < 210 g/day of red meat and < 100 g/day of processed meat

^e^Everyone consumes < 280 g/day of red meat and < 150 g/day of processed meat

## Discussion

Our study quantified that red and processed meat consumption in 2008–2009 was responsible for US$ 20.6 million in direct healthcare costs of colorectal cancer in the SUS in 2018. Considering the changes in meat consumption from 2008–2009 to 2017–2018, and the projected growing economic burden of colorectal cancer in Brazil, we estimated that red and processed meat consumption will be responsible for US$ 86.6 million in 2030. Reduced processed meat consumption to levels fixed in the counterfactual scenarios in 2030 could save US$13.7 to 74.4 million in direct healthcare costs of colorectal cancer in 2040.

Meat consumption has some adverse effects on human health [9]. Epidemiological studies have consistently reported that meat consumption increases the risk of noncommunicable diseases, such as cardiovascular diseases, diabetes, and cancer [6, 7, 26, 27]. The most robust evidence is for the harmful effect of processed meat consumption on colorectal cancer risk [6, 7]. IARC and WCRF have classified processed meat as carcinogenic to humans, whereas red meat is probably carcinogenic to humans [6, 7]. Globally, diets high in processed meats are responsible for 34 thousand cancer deaths per year, whereas diets high in red meat are responsible for 50 thousand cancer deaths per year [28]. In Brazil, red meat was responsible for 1,900 cancer cases and 1,000 cancer deaths, and processed meat for 1,700 cancer cases and 1,000 cancer deaths in 2012 [5].

Few comparative risk assessment studies have modeled the potential impact of constraining red and processed meat on health and economic outcomes, particularly on the financial burden of cancer [5, 29–31]. Our study showed that meat consumption also had escalated economic impact on the SUS over the years. In Canada, $CAN 168 million in direct ($CAN 29 million) and indirect ($CAN 139 million) costs with colorectal cancers were attributable to red meat consumption in 2014. For processed meat, these values were $CAN 202 million in direct ($CAN 34 million) and indirect ($CAN 167 million) costs of colorectal cancer in the same year [29]. In the US, the cost-effectiveness of implementing nutritional policies on processed meat consumption on the burden of cancer has been recently evaluated. The model showed that excise tax and warning labels would avert 77,000 to 85,400 cases of colorectal cancer, with net savings of $2.7 billion to $4.5 billion from a societal perspective, including $1.3 billion in healthcare costs saved [30]. These studies suggest that nutritional policies may be a cost-saving strategy that substantially benefits health and the economy. Our results showed that policies aimed at reducing processed meat consumption may significantly impact colorectal cancer costs. For instance, our projected costs saved in 2040 were 5–sevenfold higher for reducing processed meat than red meat consumption.

Changing population diets, especially foods considered “natural, normal, necessary or nice” [9, 32], is challenging. For instance, a systematic review of health-related values and preferences regarding meat consumption found that omnivores consume meat because they consider it an essential component of a healthy diet, like food, feel that meat is part of their traditions, and believe they have not the culinary knowledge and skills to prepare a proper meatless meal [33]. In addition, there is insufficient evidence of the effectiveness of interventions to reduce meat consumption [9]. The diversity of factors that influence the price and availability of meat and how it is processed and marketed determines a socioeconomic scenario that profoundly affects and is affected by norms and behavior. Changing dietary behaviors in response to interventions takes a lot of work. Still, social norms can and must change, and the coordinated efforts of civil society, health organizations, and government can aid this process. Governmental interventions could transform food systems through economic, political, and legal actions. Decreasing the production and consumption of meat and, consequently, its adverse environmental, health, social, and economic effects, as shown in our study, would be aligned with the 2030 United Nations Agenda for Sustainable Development Goals and the World Health Organization targets to reduce premature mortality from noncommunicable diseases [34]. However, the World Trade Organization rules may limit state-sponsored interventions in food systems. Recently, there has been a discussion about other interventions targeting unconscious/automatic behavioral choices for meat (e.g., choice architecture, environmental restructuring, and marketing and advertising) [9], but the effectiveness and ethics of these actions aimed at manipulating population behaviors are concerned [35]. Among the interventions proposed to reduce the consumption of red and processed meat is taxation, which has been used, e.g., as an effective tool to reduce the consumption of sugar-sweetened beverages. Efforts to raise consumer awareness of meat’s adverse health and environmental impacts, like warning labels, can also change behavior, redirecting meat subsidies to more sustainable and nutritious crops such as fruits and vegetables. Finally, national dietary guidelines can also play an essential role in decreasing red and processed meat consumption [36].

To our knowledge, this is the first study to quantify the direct costs of colorectal cancer treatment attributable to combined red and processed meat consumption. We used two nationally representative dietary survey data from over 60 thousand Brazilian adults, RR from meta-analysis, and 5.7 million nationwide registries of the SUS direct-healthcare costs of colorectal cancers. However, our study has some limitations. To estimate the PIF/PAF for Brazil, we used information on red and processed meat consumption from two 24-h real-time food records. However, we did not consider food preparations in 2008–2009 and 2017–2018, which could lead to an underestimation of meat consumption. We assumed the portability of the pooled RR from meta-analysis, which did not include Brazilian cohort studies. However, we incorporated the uncertainty of these estimates in the models via the Monte Carlo simulation approach. We also assumed at least a 10-year time lag between exposure and outcome based on the average follow-up time of prospective cohort studies [13]. We further recognize that non-linear changes concerning the cost of colorectal cancer treatment may occur. During data analysis, we tested multiple regression models by including predictor variables: time, aging, cancer incidence, cancer mortality, technological incorporation, and judicialization of health care. However, the multiple regression did not fit better than simple linear regression, and we opted for parsimony. Future modeling approaches using proportional multistate lifetable modeling of preventive interventions may be helpful to incorporate a time dimension more appropriately [37]. Our model did not account for recurring events or include the interaction between exposures and substitutions of meats from other food options.

## Conclusions

Red and processed meat consumption has an escalating economic impact on the SUS. We found that 8.4% or US$ 20.6 million in direct healthcare costs of colorectal cancer in 2018 were attributable to red and processed meat consumption. In 2030, the direct healthcare costs of colorectal cancer will increase to 19.3% or US$ 86.6 million. We estimated that reducing red meat consumption could save approximately US$ 2.2 to 11.9 million in 2040. Decreasing processed meat to levels fixed in the counterfactual scenarios in 2030 could save US$ 13.7 to 74.4 million in 2040. Our findings may help support policies to reduce meat consumption and cancer prevention strategies in Brazil. Considering the limited and finite resources available to health systems, it is urgent to prioritize primary prevention actions, especially in the context of exponential cancer treatment costs.

### Supplementary Information

**Additional file 1: Supplementary Material A.** Parameters considered in the macrosimulation model. **Supplementary Material B.** 10th revision of the International Statistical Classification of Diseases and Related Health Problems codes. **Supplementary Material C.** Relative risk of colorectal cancer per exposition category of red and processed meat consumption and sex. **Supplementary Material D.** Hyperlinks to publicly archived datasets.

## Data Availability

All data generated or analyzed during this study are included in this published article and its supplementary information files.

## Abbreviations

AICR: American Institute for Cancer Research
CUP: Continuous Update Project
CLUA: Climate and Land Use Alliance
IBGE: Instituto Brasileiro de Geografia e Estatística - Brazilian Institute of Geography and Statistics
IARC: International Agency for Research on Cancer
ICD-10: 10th Revision of the International Statistical Classification of Diseases and Related Health Problems
INCA: Brazilian National Cancer Institute
POF: Pesquisa de Orçamentos Familiares - National Household Budget Survey
PAF: Population Attributable Fraction
PIF: Potential impact fraction
PPP: Purchasing power parity
RR: Relative risks
SUS: Sistema Único de Saúde Brasileiro - Brazilian Unified Health System
SIH: Sistema de Informações Hospitalares do Sistema Único de Saúde Brasileiro - Inpatient Information System of the Brazilian Unified Health System
SIA: Sistema de Informações Ambulatoriais do Sistema Único de Saúde Brasileiro - Outpatient Information System of the Brazilian Unified Health System
WCRF: World Cancer Research Fund
