# Supplementary material for: The current and future costs of colorectal cancer attributable to red and processed meat consumption in Brazil
Source: BMC Health Serv Res. 2023 Oct 31;23:1182. doi: 10.1186/s12913-023-10169-4 (PMC10617206; doi:10.1186/s12913-023-10169-4)
Supplement: Supplementary file 1 — Additional file 1: Supplementary Material A. Parameters considered in the macrosimulation model. Supplementary Material B. 10th revision of the International Statistical Classification of Diseases and Related Health Problems codes. Supplementary Material C. Relative risk of colorectal cancer per exposition category of red and processed meat consumption and sex. Supplementary Material D. Hyperlinks to publicly archived datasets. [file 12913_2023_10169_MOESM1_ESM.pdf]

Additional file 1

Supplementary Information

The current and future costs of colorectal cancer attributable to red and processed meat consumption in Brazil

BMC Health Services Research

Leandro F. M. Rezende[1] – 0000-0003-3332-7571

Thainá Alves Malhão[2] - 0000-0002-5644-1089

Rafael da Silva Barbosa[3] - 0000-0002-9253-1487

Arthur Orlando Correa Schilithz[2] – 0000-0003-2457-3965

Ronaldo Corrêa Ferreira da Silva[2] - 0000-0002-1017-2426

Luciana Grucci Maya Moreira[2] - 0000-0003-1454-1732

Gerson Ferrari[4] - 0000-0003-3177-6576

Paula Aballo Nunes Machado[2] - 0000-0003-1693-5962

Maria Eduarda Leão Diogenes* [2,5] - 0000-0002-4134-5860

1. Universidade Federal de São Paulo, Escola Paulista de Medicina, Departamento de Medicina Preventiva, São Paulo, Brazil.

2. Instituto Nacional de Câncer José Alencar Gomes da Silva, Coordenação de Prevenção e Vigilância, Rio de Janeiro, Brazil.

3. Universidade Federal do Espírito Santo, Programa de Pós-Graduação em Política Social, Vitória, Brazil.

4. Universidad de Santiago de Chile, Escuela de Ciencias de la Actividad Física, el Deport y la Salud, Santiago, Chile.

5. Universidade do Estado do Rio de Janeiro, Instituto de Nutrição, Rio de Janeiro, Brazil.

*Correspondence author: maria.melo@inca.gov.br

List of Supplementary Materials
**Supplementary Material A:** Parameters considered in the macrosimulation model
**Supplementary Material B:** 10th revision of the International Statistical Classification of Diseases and Related Health Problems codes
**Supplementary Material C:** Relative risk of red and processed meat-associated cancers per exposition category and sex
**Supplementary Material D:** Hyperlinks to publicly archived datasets

Supplementary Material A: Parameters considered in the macrosimulation model

**Parameter**	**Variable**	**Commentary**	**Source/ Year**
Consumption of red meat	Prevalence data and median red meat consumption in adults ≥ 20 years who relied exclusively on the public health system.	We considered consumption of all types of meat from mammals, such as beef, horse, goat, lamb, mutton, and pork. We obtained the median consumption and prevalence rates for each red meat consumption category and sex. Exposition categories: <70 g/day (reference); ≥70 to <140 g/day; ≥140 to <210 g/day; ≥210 to <280 g/day; ≥280 g/day.	National Household Budget Survey – POF 2008-2009 and 2017-2018
Consumption of processed meat	Prevalence data and median processed meat consumption in adults ≥ 20 years who relied exclusively on the public health system.	We considered consumption meat preserved by smoking, curing, salting, addition of chemical preservatives (e.g., bacon, chorizo, corned beef, ham, pastrami, salami, and sausages). We obtained the median consumption and prevalence rates for each red meat consumption category and sex. Exposition categories: 0 g/day (reference); 1-<50 g/day; ≥50 to <100 g/day; ≥100 to <150 g/day; ≥150 g/day.	National Household Budget Survey – POF 2008-2009 and 2017-2018
Relative risk	Relative risk	We used the relative risks obtained from the linear dose-response meta-analysis per increment of 100 g/day of red meat consumption: 1.12 (95% CI: 1.00 to 1.25) and per increment of 50 g/day of processed meat: 1.16 (95% CI: 1.08 to 1.26). We converted	WCRF/AICR systematic review reports

		these measures per increment of 1 g/day of the exposure for colorectal cancer incidence and calculated the relative risks per exposition category considering the median consumption in each category. We stratified by sex, and exposition category.	
Direct healthcare cost	Values of inpatient procedures related to cancers in adults ≥ 30 years paid by the federal government	Federal direct healthcare costs of inpatient procedures related to cancer approved for payment in the Brazilian Unified Health System. We stratified by sex.	Hospital Information System of the Brazilian Unified Health System (SIH-SUS) 2008-2019
Direct healthcare cost	Values of outpatient procedures related to cancer in adults ≥ 30 years paid by the federal government	Federal direct healthcare costs of outpatient procedures related to cancer approved for payment in the Brazilian Unified Health System. We stratified by sex.	Ambulatory Information System of the Brazilian Unified Health System (SIA-SUS) 2008-2019

Supplementary Material B: 10th revision of the International Statistical Classification of Diseases and Related Health Problems codes

**Cancer type**	**ICD-10 code**
Colorectal	C18, C18.0, C18.1, C18.2, C18.3, C18.4, C18.5, C18.6, C18.7, C18.8, C18.9, C19, C20
All invasive cancers	C00-C97

Supplementary Material C: Relative risk of colorectal cancer per exposition category of red and processed meat consumption and sex.

**Exposition category**	**Female**	**Male**
Red meat consumption		
<70 g/day	1.00	1.00
≥70 to <140 g/day	1.03	1.03
≥140 to <210 g/day	1.13	1.13
≥210 to <280 g/day	1.21	1.21
≥280 g/day	1.38	1.43
Processed meat consumption		
0 g/day	1.00	1.00

>0 to <50 g/day	1.09	1.09
≥50 to <100 g/day	1.20	1.19
≥100 to <150 g/day	1.43	1.43
≥150 g/day	1.81	2.04

Supplementary Material D: Hyperlinks to publicly archived datasets.

**Datasets**	**Hyperlink**
Brazilian National Household Budget Survey carried out in 2008-2009 and 2017-2018	https://www.ibge.gov.br/estatisticas/sociais/rendimento-despesa-e-consumo/9050-pesquisa-de-orcamentos-?=&t=microdadosfamiliares.html?=&t=microdados
Ambulatory Information System of the Brazilian Unified Health System	ftp://ftp.datasus.gov.br/dissemin/publicos/siasus/200801_/dados
Hospital Information System of the Brazilian Unified Health System	ftp://ftp.datasus.gov.br/dissemin/publicos/sihsus/200801_/dados
